# Does Braun Enteroenterostomy Reduce Delayed Gastric Emptying After Pancreaticoduodenectomy?

**DOI:** 10.1097/MD.0000000000000048

**Published:** 2014-07-25

**Authors:** Xu-Feng Zhang, Guo-Zhi Yin, Qing-Guang Liu, Xue-Min Liu, Bo Wang, Liang Yu, Si-Nan Liu, Hong-Ying Cui, Yi Lv

**Affiliations:** Department of Hepatobiliary Surgery (X-FZ, G-ZY, Q-GL, X-ML, BW, LY, S-NL, YL); Institute of Advanced Surgical Technology and Engineering (X-FZ, X-ML, YL); Department of Chinese Acupuncture and Moxibustion, First Affiliated Hospital of Medical College, Xi’an Jiaotong University (H-YC), Xi’an, China.

## Abstract

Whether an additional Braun enteroenterostomy is necessary in reducing delayed gastric emptying (DGE) after pancreaticoduodenectomy (PD) has not yet been well investigated. Herein, in this retrospective study, 395 consecutive cases of patients undergoing classic PD from 2009 to 2013 were reviewed. Patients with and without Braun enteroenterostomy were compared in preoperative baseline characteristics, surgical procedure, postoperative diagnosis, and morbidity including DGE. The DGE was defined and classified by the International Study Group of Pancreatic Surgery recommendation. The incidence of DGE was similar in patients with or without Braun enteroenterostomy following PD (37/347, 10.7% vs 8/48, 16.7%, *P* = 0.220). The patients in the 2 groups were not different in patient characteristics, lesions, surgical procedure, or postoperative complications, although patients without Braun enteroenterostomy more frequently presented postoperative vomiting than those with Braun enteroenterostomy (33.3% vs 15.3%, *P* = 0.002). Bile leakage, pancreatic fistula, and intraperitoneal abscess were risk factors for postoperative DGE (all *P* < 0.05). Prokinetic agents and acupuncture were effective in symptom relief of DGE in 24 out of 45 patients and 12 out of 14 patients, respectively.

The additional Braun enteroenterostomy following classic PD was not associated with a decreased rate of DGE. Postoperative abdominal complications were strongly correlated with the onset of DGE. Prokinetic agents and acupuncture could be utilized in some patients with DGE.

## INTRODUCTION

Although the first series of patients undergoing pancreaticoduodenectomy (PD) was reported in 1930s, the mortality rates of this surgery was extremely high in the following 50 years (20%–40%).^1,2^ However, with advancements in surgical technique, improvements in patient selection, and progress in critical care, mortality after PD has significantly decreased over the last 3 decades, to below 3% in high-volume centers.^3–6^ Unfortunately, despite the improvements in survival rates after PD, postoperative morbidity rates remains as high as 50% nowadays even in experienced centers.^3,6,7^

Delayed gastric emptying (DGE) remains one of the major troublesome postoperative complications following PD despite continued improvements in perioperative patient management, the incidence of which ranged from 14% to 57%.^4,7–9^ DGE is generally described as prolonged retention of gastric sunction because of increased volume of decompression (800–1500 mL/d) and/or nausea and vomiting of the patients, and then resulting in delayed oral intake after surgery. Only recently has the International Study Group of Pancreatic Surgery (ISGPS) published a suggested definition of DGE after pancreatic surgery.^9^ Although it is not lethal, DGE leads to poor patient nutritional status and quality of life, and also burdens the patients and their families, as well as the hospitals, with prolonged hospital stay and increased costs.^4,7,10^ The exact causes of DGE after PD might be multifactorial but remain largely unknown, although the prior studies have speculated that comorbidities, nerve damage, altered hormonal levels, physiologic response to intra-abdominal sepsis, anastomotic techniques, and poor nutrition appear to be casual factors associated with DGE after PD.^11,12^

Surgical technical factors in the construction of gastroenterostomy have been implicated in the development of DGE.^13,14^ Braun introduced an enteroenterostomy anastomosis over 100 years ago between the afferent and efferent limbs distal to a gastroenterostomy in an attempt to divert food and bile from the afferent limb and decrease reflux into the stomach.^15^ Although the utility of Braun enteroenterostomy might decrease alkaline reflux gastritis and bile vomiting,^15^ whether a Braun enteroenterostomy is necessary in decreasing the incidence of DGE has not yet been well investigated. Two recent publications, however, with small number of patients enrolled, demonstrated that utilizing the Braun enteroenterostomy following classic PD decreased sequelae of DGE.^16,17^ Herein, the present study enrolled 395 consecutive patients undergoing PD in our institute, and aimed at evaluating possible association between Braun enteroenterostomy and DGE after PD and discussing treatments of postoperative DGE.

## PATIENTS AND METHODS

### Patient Selection

We reviewed the computed and paper records of patients undergoing classic PD and child reconstruction in our hospital from January 2009 to May 2013, and 395 consecutive patients were enrolled retrospectively. Those undergoing total PD, pylorus preserving pancreaticoduodenectomy (PPPD), or Roux-en-Y hepaticojejunostomy were excluded from the study. Two patients with a history of gastric resection were also excluded from the study. The study was approved by the Ethics Committees of the First Affiliated Hospital of Medical College, Xi’an Jiaotong University, Xi’an, China. All resected specimen were routinely examined pathologically for diagnosis of lesions, their number and size. The demographic, laboratory test, pathological examination, and postoperative morbidity were documented and compared between the patients undergoing reconstruction using a single limb of jejunum and an additional Braun enteroenterostomy with those not undergoing a Braun enteroenterostomy.

### Surgical Procedure

**FIGURE 1:**
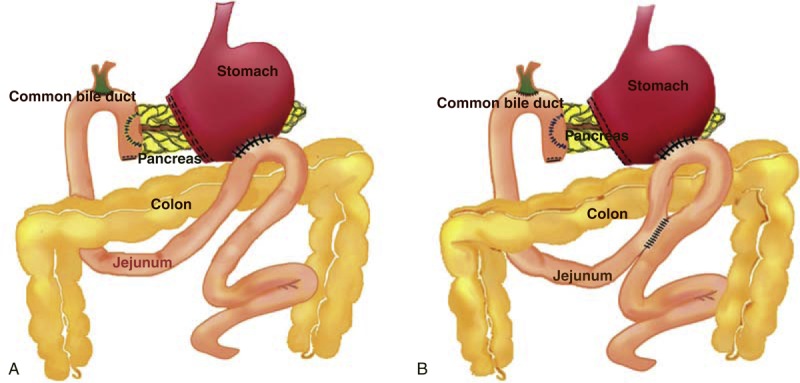
Schematic diagram of alimentary tract reconstruction (A) without Braun enteroenterostomy and (B) with additional Braun enteroenterostomy following pancreaticoduodenectomy.

All surgeries were performed by 6 well-trained senior surgeons. Selected patients underwent standard PD with antrectomy and child reconstruction, with (N = 347) or without (N = 48) Braun enteroenterostomy. Braun enteroenterostomy was routinely performed by most surgeons in our institute, while 2 surgeons preferred to do standard PD without Braun enteroenterostomy. Whether or not doing additional Braun enteroenterostomy was determined by the surgeons randomly but not based on patient condition. Child reconstruction was performed as standard procedure (Figure 1), beginning with pancreatojejunostomy (end-to-side invagination, or end-to-side duct-to-mucosa), with pancreatic duct stenting when necessary. An outer row of interrupted suture was used to approximate the pancreatic parenchyma with the jejunal seromuscular layer. End-to-side hepaticojejunostomy was performed 10–20 cm distally to pancreatic anastomosis, and antecolic or retrocolic gastrojejunostomy was performed 50 cm distally to biliary anastomosis. A Braun enteroenterostomy (Figure 1B) was created 10–20 cm distally to gastric anastomosis. Briefly, the afferent and efferent limbs of jejunum from gastrojejunostomy were brought together and anastomosed with side-to-side stapler or manual suture. The enterostomy was closed in 2 layers. One round silicon drainage tube was routinely placed posterior to the biliary and one posterior to the pancreatic anastomoses, and connected to the anti-reflux low-pressure drainage bag. A 16 F nasogastric tube was routinely positioned in the gastric fundus.

### Postoperative Management

Postoperatively, all patients stayed in the intensive care unit for 24–48 hours, unless the patients needed further monitoring because of other comorbidities or complications. Antibiotics were used prophylactically for 24–48 hours, and then ceased, unless therapeutic application was necessary. The patients were allowed to drink some fluids after passing gas and clamp of the nasogastric tube. Once the patients complained no abdominal distention or pain after orally intake of fluids for 1 day, the nasogastric tube was then removed. The patients then started to eat soft and regular diet if they could tolerate in the following days. Jejunal feeding tubes were inserted not routinely but only to those with poor general conditions and old ages (>70 y). The abdominal drainage was checked for characters and volume postoperatively, and examined for amylase if pancreatic leakage was suspected. The drainage tubes were removed if there was no evidence of any pancreatic or biliary leakage at day 5–7. No prokinetic agents were used for prophylaxis of DGE in our unit, as no prokinetic agents could reliably reduce DGE.

### Postoperative Morbidity

For comparison purpose, DGE was defined and classified by ISGPS recommendation^9^: grade A, requiring or reinserting nasogastric tube between day 4 and 7 postoperatively or the inability to tolerate solid diet by day 7 postoperatively; grade B, requiring or reinserting nasogastric tube between day 8 and 14 or the inability to tolerate solid diet by day 14 postoperatively; and grade C, requiring or reinserting nasogastric tube after day 14 or the inability to tolerate solid diet by day 21 postoperatively. Since we used to maintain the nasogastric tube for 3–7 days after passing flatus, incidence of grade A DGE was not completely recorded. It also has minor disturbances in the return to regular diet with no major clinical factors,^18^ which, therefore, was not recorded for analysis further. Pancreatic fistula was defined according to ISGPS criteria as any measurable volume of abdominal drain fluid on or after postoperative day 3 with an amylase content 3 times or greater than upper normal serum value.^7,19^ Bile leakage was defined according to the International Study Group of Liver Surgery as bilirubin concentration in the drain fluid at least 3 times the serum bilirubin concentration on or after postoperative day 3 or as the need for radiologic or operative intervention resulting from biliary collections or bile peritonitis.^10^

### Statistic Analysis

Data were expressed as mean ± standard error or median value for numerical variables, and percentages for nominal variables. Mann-Whitney U test or t tests were used to compare numerical variables, and the chi-square test or Fisher’s exact test was carried out to compare nominal variables between the groups. Statistical analysis was carried out using SPSS 17.0 (Chicago, Illinois). *P *< 0.05 was considered statistically significant.

## RESULTS

### Patient Characteristics

There were 48 patients undergoing classic PD without Braun enteroenterostomy and 347 patients undergoing classic PD with Braun enteroenterostomy during the study period. Demographics, presenting symptoms, comorbidities, and laboratory variables are shown in Table 1, and compared between the 2 groups. There was no difference in any of the baseline factors between the 2 groups (Table 1). Jaundice was the most common symptoms in most of the patients (77.2%). Preoperative biliary drainage was not routinely adopted in our patients but only to those presenting with cholangitis.

**TABLE 1:**
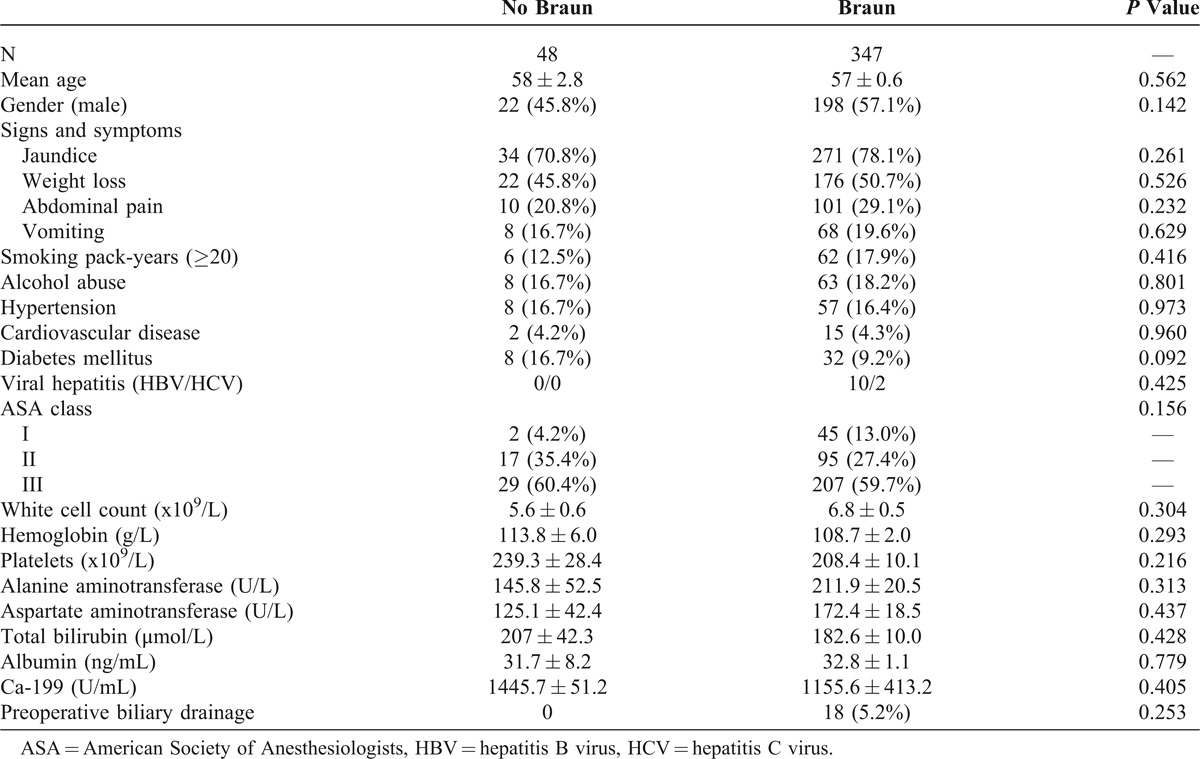
General Characteristics, Comorbidities, and Preoperative Biochemical Tests

### Operative and Pathological Variables

The pathological diagnosis of the lesions in the 2 groups is listed in Table 2. Obviously, pancreatic tumor, including pancreatic adenoma, neuroendocrine tumor, pancreatic pseudopapillary tumor, and cystic pancreatic tumor, was the most common tumor type in both the groups. There was no difference of pathological tumor types between the 2 groups (all *P *> 0.05).

**TABLE 2:**
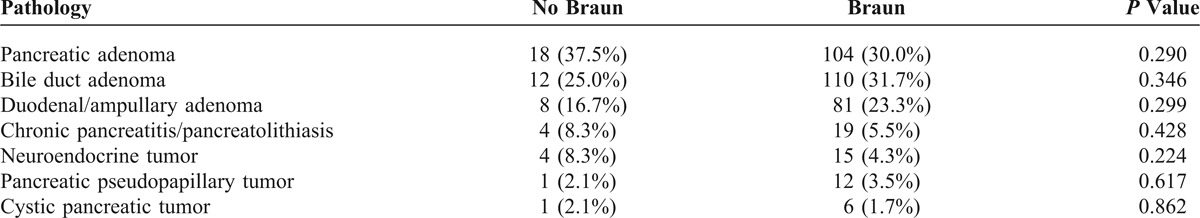
Pathological Diagnosis of the Primary Disease

The operative and postoperative details are summarized in Table 3. There was no difference between the 2 groups in tumor volume, pancreatic and bile duct size, and texture of the pancreas. However, patients with Braun enteroenterostomy less frequently underwent major vascular resection (2.3% vs 12.5%, *P *< 0.001). Intraoperative blood loss, overall stay and postoperative stay in hospital, and the total costs were similar. Although postoperative vomiting more frequently presented in patients without Braun enteroenterostomy (33.3% vs 15.3%, *P* = 0.002), the incidence of nasogastric tube reinsertion was similar between the 2 groups.

**TABLE 3:**
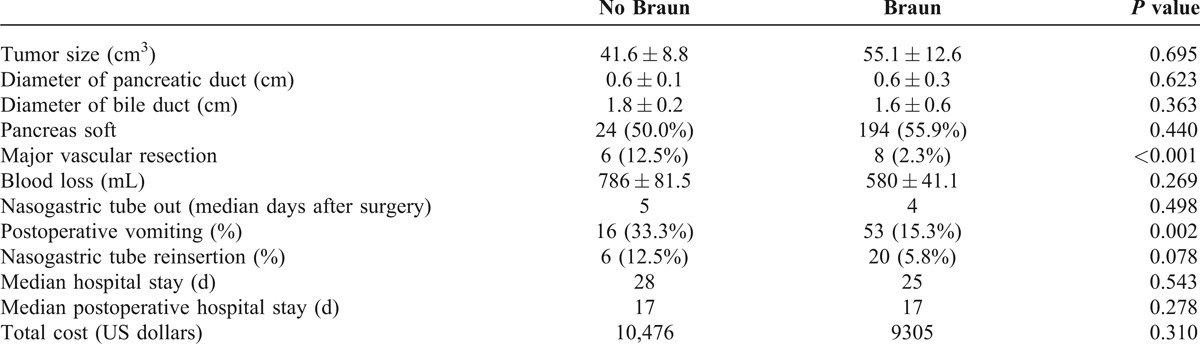
Intraoperative Findings and Postoperative Presentation

### Postoperative Complications

Postoperative complications of the patients are listed in Table 4. According to the ISGPS definition, DGE was identified in 16.7% (8/48) of the patients not undergoing Braun enteroenterostomy, but in 10.7% (37/347) of those undergoing Braun enteroenterostomy, which was not statistically different (*P* = 0.220). There was no difference in major surgery-related complications between the 2 groups, for example, bile leakage, pancreatic fistula, intraperitoneal abscess, intraperitoneal and gastrointestinal bleeding, wound infection, chylous fistula, and intestinal obstruction. However, postoperative pulmonary infection was slightly higher in patients without Braun enteroenterostomy than those undergoing Braun enteroenterostomy (16.7% vs 6.1%, *P* = 0.008). Three out of 48 (6.3%) patients without Braun enteroenterostomy underwent reoperation for intraperitoneal bleeding and/or severe bile leakage, respectively. And finally, 2 patients died of severe systemic infection and multiple organ dysfunction syndrome; 11 out of 347 (3.2%) patients undergoing Braun enteroenterostomy experienced reoperation because of intraperitoneal bleeding (5 cases), severe bile leakage (2 cases), wound dehiscence (2 cases), multiple anastomotic fistula (1 case), and intestinal obstruction (1 case). Seven patients undergoing Braun enteroenterostomy died in the hospital, and the main causes were presumed as multiple organ dysfunction syndrome (3 cases), interstitial peumonia and infection (2 cases), intraperitoneal bleeding (1 case), and cardiogenic shock (1 case). Grossly, the incidence of in-hospital death and reoperation was similar between the Braun and the no Braun groups (*P *> 0.05, respectively).

**TABLE 4:**
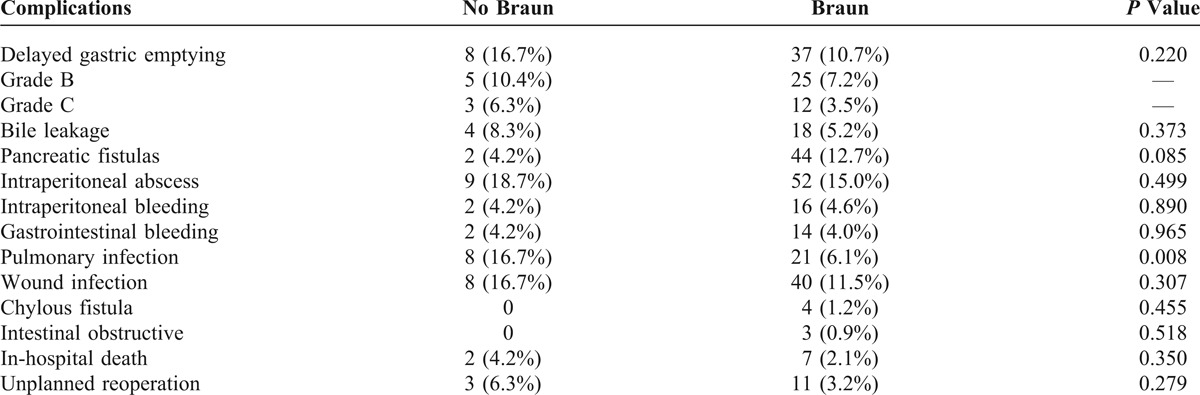
Postoperative Complications

### Factors Associated With Delayed Gastric Emptying

Various factors that might potentially be associated with DGE was investigated and analyzed by univariate analysis (Table 5). Postoperative morbidities/complications were strongly correlated with DGE, including bile leakage (odds ratio: 9.9, 95% confidence interval, 4.0–24.6) (*P *< 0.001), pancreatic fistula (odds ratio: 2.5, 95% confidence interval 1.1–5.4) (*P* = 0.019), and intraperitoneal abscess (odds ratio: 4.2, 95% confidence interval 2.1–8.3) (*P *< 0.001). However, Braun enteroenterostomy was not a risk factor associated with DGE (*P *> 0.05).

**TABLE 5:**
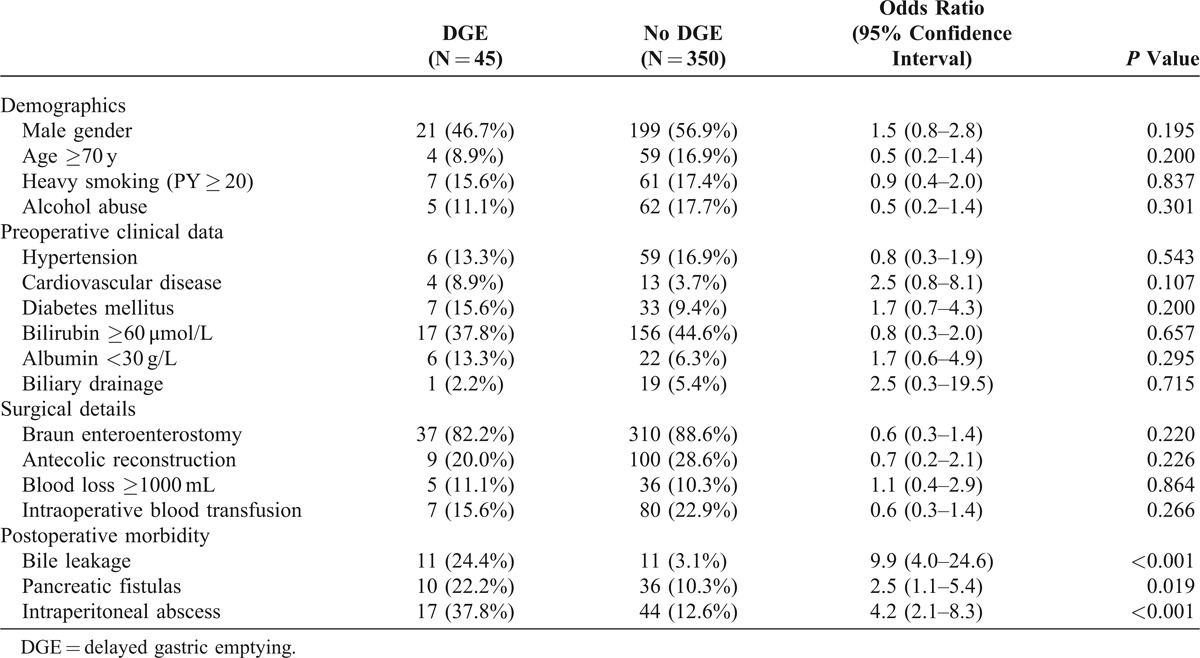
Univariate Analysis of Risk Factors Associated With Delayed Gastric Emptying

### Treatment of Delayed Gastric Emptying

**FIGURE 2:**
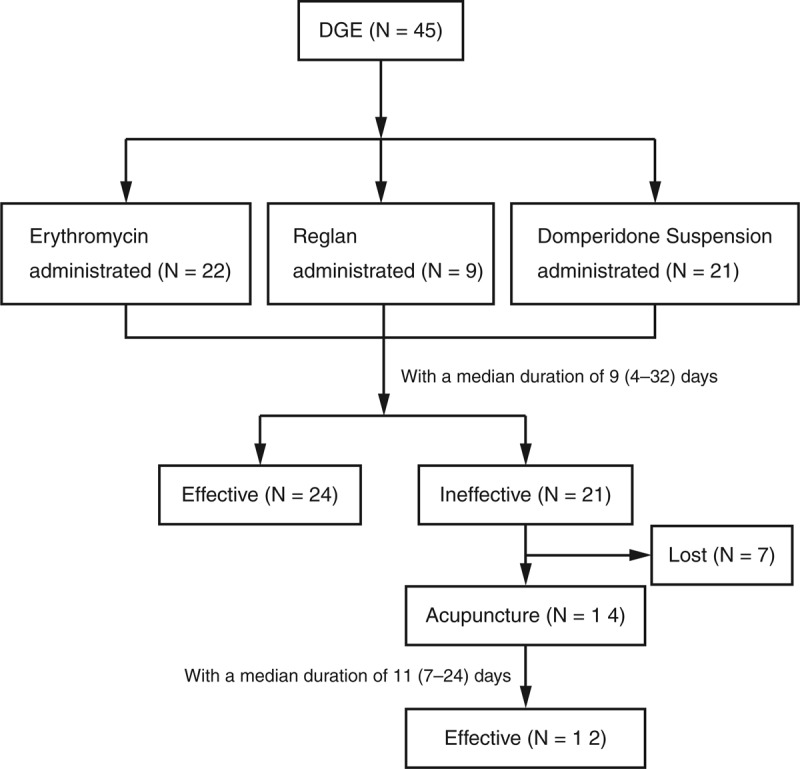
Flow chart of treatment of the patients presented with DGE after pancreaticoduodenectomy. DGE = delayed gastric emptying.

**FIGURE 3:**
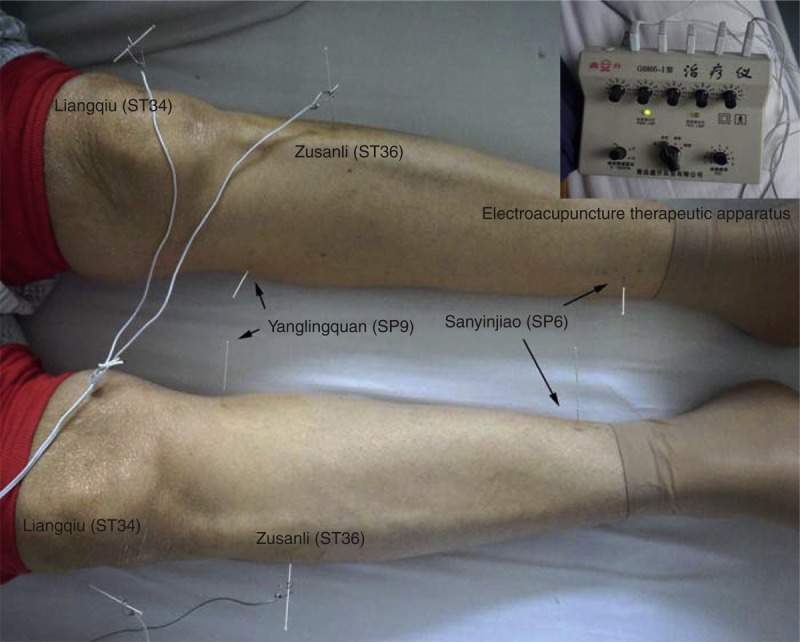
Electroacupuncture treatment of the patients presented with delayed gastric emptying after pancreaticoduodenectomy with electroacupuncture therapeutic apparatus.

Treatment of DGE is listed in Figure 2. Erythromycin, reglan, and domperidone suspension were administrated respectively or combined to the patients with a median duration of 9 days (4–32 d); 24 patients (53.3%) were finally cured with well toleration of fluid and then solid foods intake. However, the rest 21 patients (46.7%) had no obvious recovery from DGE, and then 14 of them were subjected to acupuncture. The acupoints used were ST34 (*Liangqiu*), ST36 (*Zusanli*), and SP9 (*Yanglingquan*), sometimes with the addition of SP6 (*Sanyinjiao*), all of which have certain regulation of the stomach and intestines motility from the perspective of the tranditional Chinese medicine (Figure 3). Treatments were administered twice per day for 1–3 weeks depending on relief of the symptoms. Briefly, after disinfection of the point locations and needle by 70% isopropyl alcohol, the needle was then inserted and stimulated by an electroacupuncture therapeutic apparatus (G6805-I, Qingdao Xinsheng Co, Ltd, Qingdao, Shandong Province, China) at a frequency of 2 Hz continuous waves of medium intensity. Needles were inserted bilaterally with depth of 1–2 cm and stimulated as the muscles quivered and the patient reported comfortable. The stimulating time was 30 minutes each day (Figure 3). After a median therapeutic duration of 11 days, 12 out of the 14 patients were relieved significantly in symptoms of DGE.

## DISCUSSION

DGE is a common disorder following upper abdominal surgery, especially after pancreatic and gastric resection. The incidence of DGE after PD may occur in 14%–57% of patients.^4^ This wide range of reported DGE in different centers might be in part explained by difference in definition of DGE and perioperative treatment of patients. In 2007, ISGPS recommended the universal definition of DGE with 3-grade classification.^9^ This definition has been validated and adopted by many high-volume centers. Our study indentified DGE (grades B and C) after PD by ISGPS classification was 11.4%. In our study, we did not consider DGE of grade A, since our postoperative protocol following PD was to maintain the nasogastric tube once the patient has experienced passing flatus and no abdominal distention or pain after orally intake of fluids, usually lasting for 3–7 days postoperatively. And also grade A DGE has minor disturbances in the return to regular diet and no impact on postoperative course or duration of hospital stay.^18^ Thus, taking grade A DGE into consideration, the incidence of DGE in our study must be higher than 11.4%.

The pathophysiology of DGE is probably multifactorial and has been thought to be associated with: disruption of vagal and sympathetic innervation to the antropyloric regions,^20,21^ absence of hormonal stimulation (motilin) due to resection of the duodenum,^22,23^ gastroparesis secondary to abdominal complications,^24,25^ torsion or angulation at the gastroenterostomy related to anastomotic techniques,^17^ and poor general condition and comorbidities of the patients preoperatively.^11,12^ Moreover, surgical techniques have also been reported and associated with postoperative DGE. DGE after PD has been attributed to pylorus preservation,^26^ however, some other studies have suggested no marked difference in DGE between patients undergoing PPPD and traditional PD.^24,27^ Whether an additional Braun enteroenterostomy would necessarily decrease postoperative PD remains undetermined. Two recent studies suggested that Braun enteroenterostomy was associated with decreased incidence of DGE following classic PD.^16,17^ The theoretical explanation was that Braun enteroenterostomy allows a shortcut between afferent and efferent limb, which would avoid the increase of pressure in the biliopancreatic limb and reduce bile reflux gastritis.^16,17^ Although it might cause early postoperative vomiting, bile reflux gastritis is not definitely associated with postoperative DGE. In our study, patients undergoing PD without Braun enteroenterostomy had higher incidence of postoperative vomiting than those undergoing PD and Braun enteroenterostomy (33.3% vs 15.3%, *P* = 0.02). However, the incidence of DGE after surgery was not significantly different between the 2 groups by ISGPS criteria (16.7% vs 10.7%, *P* = 0.220). We regularly maintained the nasogastric tube for 3–7 days until the patient could tolerate liquid diet for 1 day after clamping of the tube. Therefore, the longer retention of the nasogastric tube might attenuate the alkaline reflux gastritis and its influence on gastric mobility by extracting bile and pancreatic juice refluxed into the stomach at early stage. In our study, we enrolled 395 patients undergoing PD in our center from 2009 to 2013. Although 6 surgeons performed the procedures, all followed identical standardized perioperative and operative protocols. The decision not to do Braun enteroenterostomy was made by the surgeons randomly but unrelated to patient condition. Although there might be a potential selection bias, the patients undergoing PD with and without Braun enteroenterostomy were not different in patient characteristics, preoperative comorbidities, or general conditions. And also, pathological diagnosis, lesion status, surgical procedure, postoperative treatment, or most complications were not different between the 2 groups either. Therefore, the patients in the 2 groups with and without Braun enteroenterostomy following PD had comparable baseline characteristics. However, we noticed major vascular resection was more common in patients without Braun enteroenterostomy than those with Braun enteroenterostomy (12.5% vs 2.3%). The reason was, however, unclear. A selection bias might be possible that surgeons tended to shorten the operation time by not doing Braun enteroenterostomy following PD after vascular resection and construction.

We also examined potential risk factors contributing to DGE. Braun enteroenterostomy was not correlated to occurrence of postoperative DGE following PD. It was consistent with the previous studies^25,28^ that postoperative abdominal morbidity, such as bile leakage, pancreatic fistulas, and intraperitoneal abscess, was strongly associated with postoperative DGE in our study. The reason why postoperative abdominal morbidity might lead to DGE remains obscure. It has been suggested that abdominal inflammation or abscess might directly cause gastrointestinal paralysis.^25,28^ Therefore, DGE can be regarded as a surrogate marker for postoperative complications and hence should be closely watched for.

As no prokinetic agent has reliable effect on prevention or management of DGE, we did not routinely use these agents prophylactically. The role of erythromycin in the management of DGE has been investigated in some randomized controlled trial (RCT) studies, the results, however, were controversial.^29–32^ The insufficient effects of prokinetic agents might signify the multifactorial causes of DGE. Mostly out of experience but not evidence, we regularly use prokinetic agents for management of DGE. In our study, DGE symptoms of some patients (24/45, 53%) were significantly relieved after a median duration of 9 days use of erythromycin, reglan, and/or domperidone suspension. As an ancient Chinese traditional method, acupuncture has been found to affect acid secretion, gastrointestinal motility, and neurohormonal leves, and improves gastroparesis symptoms.^33–36^ And it has been also shown that acupuncture could relieve dyspeptic symptoms of patients with diabetes and accelerate gastric emptying.^35,37–39^ In the present study, electroacupuncture was used for 14 patients who were unresponsive to prokinetic agents for a median duration of 11 days, and 12 of them had a significant relief of the symptoms. The mechanism of acupuncture in the improvement of gastrointestinal motility and symptoms is largely unkown. From the perspective of traditional Chinese medicine, ST34, ST36, SP6, and SP9 are all correlated to gastrointestinal motility, and stimulation of these acupoints has shown to excite vagal activity and enhance gastric myoelectric activity.^36,39,40^

## CONCLUSION

In this retrospective series, the addition of Braun enteroenterostomy following classic pancreaticoduodenectomy was not associated with a decreased rate of DGE or other complications. Therefore, postoperative abdominal complications were strongly correlated with the onset of DGE. The use of prokinetic agents and acupuncture might improve symptoms of DGE in some patients. However, because of the limitation of this retrospective study in a single institution, an RCT should be further conducted to evaluate the effects of Braun enteroenterostomy on the incidence of DGE, and also the efficacy of the treatments available.
